# People’s Experience of Shared Decision Making in Musculoskeletal Physiotherapy: A Systematic Review and Thematic Synthesis

**DOI:** 10.3390/bs12010012

**Published:** 2022-01-12

**Authors:** Jessica Grenfell, Andrew Soundy

**Affiliations:** 1 Physiotherapy, Cornwall Partnership NHS Foundation Trust, Bodmin Community Hospital, Boundary Road, Cornwall PL31 2QT, UK; 2 School of Sport, Exercise and Rehabilitation Sciences, University of Birmingham, Birmingham BL15 2TT, UK

**Keywords:** shared decision-making, decision-making, patient involvement, patient experience, musculoskeletal physiotherapy, communication

## Abstract

(1) Shared decision making (SDM) has been advocated as a way of improving prudency in healthcare and has been linked to self-efficacy and empowerment of service users. The evaluation of its use in musculoskeletal (MSK) physiotherapy has been vague, but articles suggest that trust and communication are integral. (2) ENTREQ guidelines informed this systematic review and thematic synthesis. PRISMA recommendations steered a systematic literature search of AHMED, CINAHL, MEDLNE, EMBASE and Cochrane databases from inception to September 2021. COREQ was used for quality appraisal of articles alongside critical discussions. Analysis and synthesis included five stages: outlining study characteristics, coding of data, development of descriptive themes, development of analytical themes and integration and refinement. The review aim was to explore people’s experiences of SDM in MSK physiotherapy and to inform our understanding of the conditions needed for successful SDM. (3) Out of 1508 studies, 9 articles were included. Four main themes (trust, communication, decision preferences and decision ability) demonstrated that the majority of people want to participate in decision-making. As described in the capacity and capability model, three core conditions were needed to facilitate someone’s’ ability to participate. (4) People want to be involved in SDM in MSK physiotherapy. For successful SDM, physiotherapists should look to develop mutual trust, utilise two-way communication and share power.

## 1. Introduction

Shared decision making (SDM) can be understood by fundamental values [1] rather than an agreed definition [2]. Three principles have been identified: (a) a collaborative relationship between healthcare professionals and those accessing healthcare, including their caregivers [2], (b) a recognition that both parties influence the decision-making process [2,3] and (c) the values and preferences of the person accessing healthcare should be central to decision-making, underpinned by support to allow an informed understanding of the available options [4,5].

SDM has long been advocated by policy makers [6,7] to facilitate prudent healthcare [8] and reduce health inequalities [9]. SDM can positively impact people’s satisfaction of healthcare [10,11] and may also be linked to deeper concepts such as self-efficacy, autonomy and empowerment [2,12]. While research into SDM has grown exponentially in recent years [12], the majority is focussed on primary care [1]. Further research is needed on its use in other areas including physiotherapy [13], particularly in underserved specialities such as musculoskeletal (MSK) physiotherapy.

Research that has been published in this area often focusses on clinician viewpoints or observer perceptions as opposed to the perspective of patients [13,14,15], despite the suggestion that understanding public views is essential if SDM is to be fully embedded in healthcare [16]. Initial research that has focussed on MSK physiotherapy patients has shown that SDM may enhance trust, satisfaction and empowerment to participate in decision-making [17,18], but these findings are vague and varied [18,19,20]. Other studies use quantitative methods to investigate this phenomena [21], which may misrepresent findings and therefore our understanding of this complex phenomena [21,22]. Analysing the available data in a systematic review would provide clarity and an opportunity to identify associations between themes and concepts into a model, beyond the understanding of the original studies [23]. To the best of the authors’ knowledge, there are no current reviews that have done this.

Given the above, the aim of this review is to systematically search and thematically synthesise peoples’ experiences of SDM in MSK physiotherapy, to understand the conditions needed for successful SDM.

## 2. Materials and Methods

A subtle realist paradigm informed the review’s approach [24]. This allowed for subjective exploration and understanding of experiences, which could go on to have implications for the wider population [25]. A thematic synthesis was undertaken [23], which involved 3 phases:

1. A systematic search and data extraction;
2. An appraisal of qualitative literature;
3. A synthesis of qualitative data.

The review was written in line with the ENTREQ guidelines [26].

### 2.1. Literature Search

Following a scoping literature review [27], a comprehensive, pre-planned systematic literature search was undertaken up to 8 September 2021 [28]. Electronic databases including AHMED, CINAHL, MEDLNE, EMBASE and Cochrane were searched using key terms pertinent to the phenomena and population in question. Search terms were translated from the research question [29] and informed by the eligibility criteria. They were ‘shared decision making’, ‘person centred care’, ‘patient centred care’ and ‘physiotherapy’ or ‘physical therapy’. Standard Boolean and proximity operators including ‘AND’, ‘OR’ and ‘NEAR’ were employed.

Targeted reference list checking of systematic reviews in related areas was completed [30,31], as well as reference list checking of included articles. Searches using Google Scholar and FinditBham search engines, and Grey literature searches of relevant websites including the Chartered Society of Physiotherapy, Choosing Wisely and the Health Foundation were also completed. The key authors’ catalogues of publications were also explored. Titles and abstracts were screened for relevance, then full text articles were scrutinised for their eligibility (see Supplementary File S1).

Eligibility criteria was refined using the SPIDER acronym [32].

- S: individuals who have experienced MSK physiotherapy. Studies relating to other specialities within physiotherapy were excluded.
- PI: studies must have included at least 1 paragraph of explicit reference to SDM. Articles were accepted if this was contained within broader phenomena such as person-centred care or satisfaction. Articles were accepted as referencing SDM when the following could be identified, as discussed in the principles of SDM above: (a) a relationship between physiotherapist and person accessing physiotherapy, (b) a collaborative approach to discussions about rehabilitation options and (c) a discussion of the person’s preferences for rehabilitation.
- D: a broad range of qualitative types were included, whilst quantitative research, conference proceedings and pilot studies were excluded.
- E: exploration of people’s experiences of SDM in MSK physiotherapy. Studies must report first-hand experiences of people, as opposed to perceptions of healthcare professionals. If an article included both perspectives, clear distinction between clinicians’ and people’s views was required.
- R: all qualitative research types were included in the search. As SDM is a complex intervention likely affected by multiple factors [12], the intention to fully understand context is integral, and quantitative research limited to numerical data may misrepresent findings [22]. Moreover, as the aim of this review is to understand the patient’s perspective, retaining narratives presented through qualitative research is fundamental, resulting in the exclusion of quantitative methodologies.
- Other: date of publication was not limited, as a scoping literature search demonstrated no known systematic reviews published previously. Language was limited to English as the primary language understood by the review team.

### 2.2. Study Selection and Data Extraction

Author JG searched for articles and applied the eligibility criteria during the title and abstract screening. If the eligibility criteria were not clear, full texts were retrieved and screened. The included articles were reviewed by JG, where data extraction was performed and recorded in a standardised form (see Table S1).

### 2.3. Critical Appraisal

While determining quality in qualitative research remains contentious [33], reviewing whether research has been explicit and transparent in its reporting is still useful in determining trustworthiness [33]. The consolidated guidelines for reporting qualitative research (COREQ) were used [34] to inform the quality assessment. This has questions split into 3 domains, resulting in a score out of 32. Author JG completed the COREQ for each study, and any articles that scored lower than 20 were critically discussed with author AS to assess their trustworthiness and value (see Supplementary File S2).

### 2.4. Analysis and Synthesis

Analysis and synthesis consisted of 5 stages. The first involved identifying study characteristics and participant demographics (see Table S2). The second stage involved coding of all relevant data from the studies’ results and discussions. Mind mapping was used to develop and group descriptive themes in stage three, where data was grouped inductively, and novel themes were created iteratively where needed [22] (see Table S3). Analytical themes were then developed through integration of contextual factors, in order to develop novel concepts, which built on the preliminary studies [35] in stage 4 (see Table S4), and thematic refinement and integration occurred in stage 5 (see Supplementary File S3).

## 3. Results

**Figure 1:**
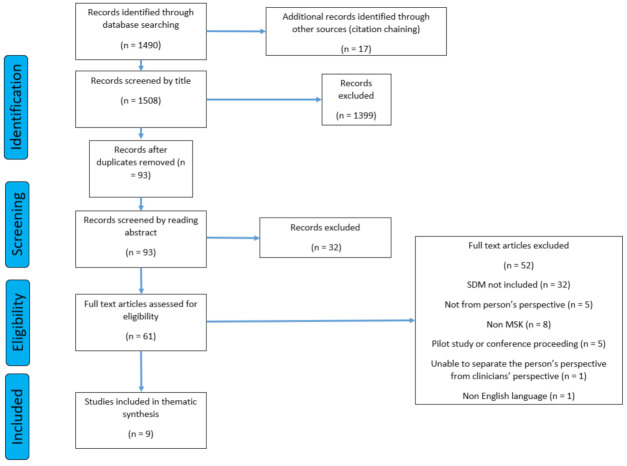
The PRISMA flow diagram.

Of the 1508 articles found, 1499 were excluded, meaning 9 studies were included. Despite including all design types in the literature search, all included studies were of qualitative design as a result of the remaining eligibility criteria. The full search strategy is represented in the PRISMA diagram (Figure 1) [36].

### 3.1. Study Characteristics

Two hundred and thirty-two participants were included across the nine studies. One study did not comment on age [37], but those that did demonstrated a range between 18 to 81 years old. Four studies did not report symptom duration [37,38,39,40], but those that did reported a range of less than three weeks to 40 years. Education background and employment status were varied throughout the studies. Eight of the studies took place in the USA and Europe [19,37,38,39,40,41,42,43] and one took place in Egypt [20].

### 3.2. Critical Appraisal

The quality of the studies was varied. Those that had a COREQ score under 20 [19,37,39,40] generally did so because they did not report on reflexivity and transparency. However, during critical appraisal discussions, the authors felt that as the focus of this review was on data development and conceptual saturation [44], a greater weighting was placed on assessing how robust the approach to data analysis and synthesis was, with less weighting on reflexivity. Despite the low COREQ scores, all studies were deemed by the authors to have trustworthy, plausible results [19] or provide a unique insight into thematic synthesis [37] and so were included in this review (see Supplementary File S2).

### 3.3. Synthesis

Four main themes were identified with eight subthemes. The development of mutual trust, two-way communication, and a collaborative approach to sharing power all facilitated SDM and is discussed further below.

### 3.4. Theme 1: Trust

The development of trust was cited by almost all of the studies. It resulted in both positive and negative outcomes in relation to SDM.

#### 3.4.1. The Development of Trust

People felt that trust was fostered by perceived passion, personal competence, communication skills and empathic personality traits demonstrated by the physiotherapist [20,38,39,41,42,43]. Trust could be fostered solely because the physiotherapist was deemed an ‘expert’ [20,40,43].

#### 3.4.2. The Positive Impact of Trust

Trust often resulted in a positive experience and could be mutual [37], also improving engagement [42] and reducing fear [37,41]. People felt the physiotherapist would choose what was best for the individual [20,37,43], especially if they were unsure of their own preferences [43].

#### 3.4.3. The Negative Impact of Trust

Unilateral trust in the physiotherapist was cited as a reason to defer involvement in SDM [19,20,37,38,42,43]. Some were content that the ‘expert’ physiotherapist would make the right decision [38,40,42]. One participant commented that the ‘therapist knows best’ (p. 216, [37]). However, this perceived ‘expertise’ was not always welcomed and ‘know it all’ physiotherapists misunderstanding people’s preferences led to dissatisfaction and an inability to participate in SDM (p. 217, [37]).

### 3.5. Theme 2: Communication

Communication was common across all studies in relation to SDM. People needed information provision from the physiotherapist and to be listened to, meaning two-way communication was essential.

#### Two-Way Communication Is Essential for Collaboration

People wanted information on diagnosis, prognosis, treatment and self-management strategies [19,20,38,40,41,42,43]. It had to be presented in an understandable way [41] because when it was not, people reported a negative experience [20,38,40]. Conversely, being offered appropriate information led to a positive experience [38,40,43], the ability to have fears allayed [19] and empowerment to make informed decisions [20,43]. People felt it was integral to have their preferences heard [37,41,42,43] even if they were divergent to the physiotherapist’s [42]. Decisions should not be made without being listened to [42] and a therapist demonstrating empathy resulted in greater satisfaction and trust [38,40,41,43]. Being listened to enabled people to participate in decision-making [43] and scenarios that actively encouraged questions facilitated this [38,43]. Collaborative communication was integral for a good experience [40], whereas didactic communication was perceived negatively [37,40].

### 3.6. Theme 3: Decision Preferences

Across the studies, people’s motivation to be involved in decision-making varied. Whilst some wanted involvement, others did not, and the reasons for this were multifactorial.

#### 3.6.1. Preferences for Involvement in Decision-Making

Preference for involvement in SDM varied [19,37,38,42,43]; some people were passive and some completely autonomous but the majority wanted to share responsibility for decision-making to some degree [37,42]. Some people wanted to be involved in decision-making throughout the rehabilitation process [20,37,42,43] even if they did not make the actual decision [42]. Some wanted their preferences to be taken into account [37,40] and wanted choice [38]. Some people were empowered to collaborate and make decisions [42], and someone said if they could have made all the decisions they would have [37]. In contrast, some were happy to defer decisions to the physiotherapist [19,20,38,42,43]. Satisfaction with involvement was divided; some were happy with their level of involvement in decision-making, whilst others were dissatisfied [37].

#### 3.6.2. Factors Which Influence Involvement

Empowerment enabled people to participate in decision-making [42]. People’s preferences for involvement could vary based on each decision, and this could be due to the perceived level of associated risk [19,37]. Reasons for opting out of SDM included the perception that the physiotherapist was the expert and knew the person’s preferences [19,20,38,42], because of the fear of making the wrong decision [19,20], if the explanation of the option was good [38] and if the decision was ‘minor’ [19]. Some felt it was the clinician’s role to make the decision in the best interest of the person, and the person’s role to listen [19,20,37,38,42]. One study showed variation could be due to cultural, social and economic factors [37].

### 3.7. Theme 4: Decision Ability

The ability to participate in SDM was affected by individual experience, confidence and knowledge, as well as the environment created by the physiotherapist.

#### 3.7.1. People Are Not Involved in Decision-Making

Some physiotherapists laid out options then chose what they thought best [42] or chose the treatment completely independently of the person [19,37,40,43]. Other times, there were no or only some treatment options laid out [38,43]. At times, the physiotherapist ignored the person’s preferences entirely, leading to a negative, sometimes emotional, experience [38]. Some said if they had more involvement, it could have improved concordance with a program [37,38,40].

#### 3.7.2. The Power Struggle

More knowledge and experience resulted in a greater ability to participate in decision-making and to self-manage [19,20,38,42,43]. Positive previous experience led to increased self-confidence [40,43] and a greater ability to ask questions [19,40,43]. Lack of confidence led to an inability to challenge the physiotherapist when more involvement was wanted [19]. Knowledge could be gained from information provision from the therapist leading to empowerment [20,42,43], whilst a perceived lack of knowledge left people unable to challenge the physiotherapist, and unable to help themselves [19]. Negatively in some cases, physiotherapists exerted ‘power’ over the relationship [40] and people were told what to do [19]. If this opposed the person’s beliefs, it impacted the experience negatively [19,38,40].

The results of this review depict three central conditions that impact someone’s capacity and capability to participate in SDM in MSK physiotherapy. The three conditions were: (a) mutual trust, defined as reciprocal confidence in both parties’ abilities and expertise, (b) two-way communication, categorised as information sharing between both parties where the physiotherapist imparts knowledge, and the person voices their preferences and (c) sharing power, where the clinician relinquishes sole control over the relationship to allow the person to actively engage in decision-making.

**Figure 2:**
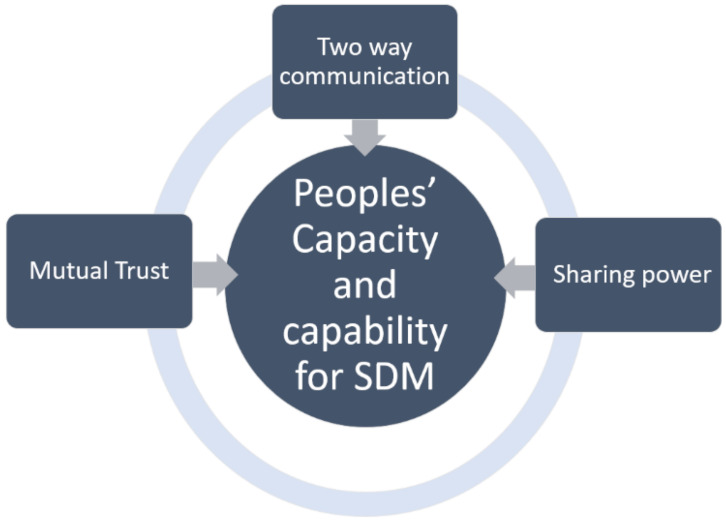
The capacity and capability (CAC) model for enhanced SDM.

The cyclical model above (Figure 2) describes the central conditions impacting someone’s ability to participate in SDM, but also demonstrates the interrelationships between the conditions. Two-way communication enabled people to challenge the physiotherapist to share power [20,42,43] and empathic communication strategies used by the physiotherapist helped to positively develop trust [20,38,39,41,42,43]. This, in turn, allayed people’s fears, and made them more likely to participate in SDM. Although split into distinct themes, each condition benefitted from the presence of the next.

## 4. Discussion

As the first systematic review and thematic synthesis in this area, this study shows new understanding into the experiences of SDM in MSK physiotherapy, and also gives novel insight into the barriers and facilitators of successful SDM in this setting, directly from those experiencing it. Most people want to be involved in decision-making, and in order to achieve collaboration, people need the capacity to participate and confidence in their own skills. Both can be cultivated as a result of mutual trust, the presence of two-way communication and a willingness from the physiotherapist to share power within the relationship.

### 4.1. Trust

Trust was fostered in the physiotherapist due to personality traits and competence, but also because the clinician was perceived to be an expert. This phenomenon is mirrored across healthcare [45] and has been shown to be both a facilitator and barrier to SDM [31]. Trust in the clinician can improve people’s confidence to participate in SDM but can also lead people to defer decision-making to the expert [16]. The development of mutual trust, where the person is encouraged to recognise their own expertise, may well negate the negative impact that unidirectional trust in the clinician can have on influencing people to defer decision-making [31]. Furthermore, the notion that a healthcare professional knows best [46], and the desire for people to conform to societal norms about how a ‘good’ patient behaves, are well documented phenomena [47,48]. Studies have even shown that people fear the quality of care will be affected if their beliefs diverge from a doctor’s [49]. However, the current results reveal that some people resisted a perceived need to conform, which could represent dissatisfaction with traditional patient roles in MSK physiotherapy.

### 4.2. Communication

Information provision is essential for effective collaboration to enable people to participate in unfamiliar forums [16,50,51]. In this review, appropriate, understandable information allayed people’s fears and empowered them to make decisions, highlighting the need for clinicians to share knowledge in an accessible way if collaboration is the goal [2]. However, unidirectional information provision alone is not infallible. In this review, two-way communication, which allowed people to be listened to and have their preferences impact decision-making, had wider reaching benefits; not only did it facilitate SDM, it also improved satisfaction and fostered mutual trust. Improved quality of care resulting from a person-centred approach has been previously evidenced [41] and environments that support people to ask questions have been shown to be integral for SDM [52,53]. Overall, physiotherapists should continue to engage and activate the public [54], not only to facilitate SDM, but to ensure a positive therapeutic experience [38,39,43].

### 4.3. Decision Preferences

In this review, the desire to participate in SDM was individual, with some wanting to be involved and others wishing to defer decisions. This reflects decision-making preferences elsewhere in healthcare, where the majority of people want to participate and fewer wish to opt out [55,56], suggesting common values and behaviours towards SDM. In this review, preference for involvement in decision-making could vary with each decision [20,38], meaning that flexibility and reflexivity is needed from both parties for successful SDM [16].

Reasons for wishing to defer decision-making to the physiotherapist were broad. Fear of making the ‘wrong’ decision was cited [19,20], suggesting that people are more likely to opt out of high-risk decisions, although other participants were happy to delegate ‘minor’ decisions [19]. Comparatively, a study found that ‘significant decisions’ around cancer treatments could be both a barrier and facilitator to participation in SDM [57]. This means that instead of being solely risk sensitive, decision preference is personal, and likely based on individual values. Fear of making the wrong decision could also come from a perception that the person lacks medical knowledge, especially when compared with a clinician [46,58]. To negate this, people need awareness of the expertise they bring in terms of their preferences, values and beliefs, which is dependent on the clinician highlighting this [31]. Additionally, fear of making the wrong decision also implies that if the decision is deferred to a physiotherapist, the clinician would have responsibly for a potentially negative outcome. Instead, the attitude needs to move towards an acceptance that decisions are rarely good and bad, but the most appropriate decision for that person at that time [45], especially given that treatments that are 100% successful and 100% side effect free rarely exist in MSK physiotherapy.

One study showed decision preference may be impacted by cultural, social and economic factors [37], which is also reflected in other healthcare settings [31]. Whilst these demographics are fixed, the resultant behaviour has been shown to be modifiable if a person is offered the right decision support [31]. Therefore, regardless of background, people have the capacity to change their attitudes and behaviours towards SDM, if the appropriate support is provided.

### 4.4. Decision Ability

People were often prevented from participating in SDM by the physiotherapist and elsewhere in healthcare, clinicians have been known to present options in an inherently biased way [47]. Importantly, it may then not be true to say that people do not want to participate in SDM in MSK physiotherapy but that they cannot, something which is echoed in other settings [31]. Whilst collaboration has been shown to be challenging between people and physiotherapists [30], for SDM to occur, dedicated clinicians need to facilitate the sharing of power [52].

These results show that some felt the physiotherapist exerted excessive power over the relationship, resulting in a didactic, paternalistic approach which was negatively received, and has been shown to block participation in SDM [47]. This may be due to the clinician seeing their role as the decision-maker, acting as an advocate for their patients [16]. Another study demonstrated that physiotherapists often misjudge people’s preferences for involvement in decision-making [21], denoting that whilst often well-meaning, therapists might avoid using SDM due to the assumption that patients do not want to participate [16]. For collaboration to occur, there needs to be a change in clinician attitudes and behaviours.

As well as the physiotherapist sharing power, there is also a need to build people’s capacity and capability to participate in SDM. In this review, a lack of knowledge and confidence left people unable to challenge the physiotherapist and unable to help themselves, resulting in dependency and disempowerment. An inability to participate due to lack of information, confidence or an absence of an environment that encourages collaboration, should not be confused with not wanting to participate [18,59]. This could be as simple as giving explicit permission for people to be involved [46] or as complex as challenging attitudes and behaviours at the individual and societal level [60].

### 4.5. Limitations

There is the likelihood of language bias as a result of limiting the search to English language studies, which may limit the applicability of findings outside of English-speaking settings.

There is always a need to consider subjectivity and bias in qualitative research, and whilst the authors’ approach to analysis and thematic synthesis was systematic [61], it is still individual and subject to personal values and beliefs. However, the aim of this review is to create new interpretations and perspectives of findings from previous studies [62], instead of claiming that the synthesis of those findings is superior to the studies themselves [63]. Throughout the process, the authors maximised reflexivity and transparency by using collaborative reflection, reflexivity logs and audit trails [64] (see Supplementary File S4).

The quality of the studies included in this review was judged to be varied. Some had low COREQ scores, generally due to an absence of reporting on research team reflexivity [19,37,39,40]. This lack of transparency in subjectivity should make the reader challenge the credibility of the research findings [65] and could therefore have an effect on the trustworthiness of the findings of this review. However, none of the low scoring studies demonstrated any findings that were anomalous to the other studies included, allowing for a degree of confidence in their results, despite having methodological flaws.

### 4.6. Implications

The results of this review highlight clear conditions that influence people’s capacity and confidence to participate in SDM in MSK physiotherapy. The development of mutual trust, two-way communication which facilitates the sharing of information and allowing people to be heard, and the sharing of power within the relationship are all conditions which enable people to participate in decision-making. If SDM is the goal, physiotherapists have a responsibility to address these conditions utilising open and empathic communication strategies alongside approaches which look to increase people’s activation. Future research should focus on establishing how these approaches work best in MSK physiotherapy; this could be through exploration of accepted SDM models [66] or through novel approaches which relate to the specific relationship and contextual setting.

## 5. Conclusions

This review provides a novel perspective of people’s experiences of SDM in MSK physiotherapy and shows that, generally, people want to be involved in decision-making. It demonstrates people’s perceptions of the key conditions required if SDM is to be successful. The main barriers to collaborative decision-making were a lack of opportunity, confidence and capability on the person’s part, which were impacted by the attitudes and behaviours of the physiotherapist. For successful SDM, clinicians need to provide decision support through two-way communication, sharing their expertise in an understandable way, and listening to and acting on peoples’ preferences. Mutual trust needs to be developed to enable people to feel comfortable to participate. Lastly, physiotherapists must be aware of their influence as the healthcare professional and accept responsibility to create an environment that actively encourages peoples’ participation, self-efficacy and empowerment, through the sharing of power.

## Data Availability

The Supplementary File includes data that was used throughout the stages of analysis. Full data is obtainable from the original articles.

## Supplementary Materials

The following are available online at https://www.mdpi.com/article/10.3390/bs12010012/s1, Supplementary File S1: Search strategy audit trail, Supplementary File S2: Critical Appraisal discussions, Supplementary File S3: Analysis, synthesis and refinement, Supplementary File S4: Reflexivity Biography, Table S1: Data extraction, Table S2: Study characteristics, Table S3: Descriptive themes, Table S4: Analytical themes.

## References

[1] Légaré F., Adekpedjou R., Stacey D., Turcotte S., Kryworuchko J., Graham I.D., Lyddiatt A., Politi M.C., Thomson R., Elwyn G. (2018). Interventions for increasing the use of shared decision making by healthcare professionals. Cochrane Database Syst. Rev..

[2] Légaré F., Stacey D., Pouliot S., Gauvin F.P., Desroches S., Kryworuchko J., Dunn S., Elwyn G., Frosch D., Gagnon M.P. (2011). Interprofessionalism and shared decision-making in primary care: A stepwise approach towards a new model. J. Int. Care.

[3] Towle A., Greenhalgh T., Gambrill J., Godolphin W. (1999). Framework for teaching and learning informed shared decision making. Competencies for informed shared decision making. Proposals based on too many assumptions. BMJ.

[4] Elwyn G., Edwards A., Kinnersley P. (1999). Shared decision-making in primary care: The neglected second half of the consultation. Br. J. Gen. Pract..

[5] Towle A., Bainbridge L., Godolphin W., Katz A., Kline C., Lown B., Madularu I., Solomon P., Thistlethwaite J. (2010). Active patient involvement in the education of health professionals. Med. Educ..

[6] Härter M., van der Weijden T., Elwyn G. (2011). Policy and practice developments in the implementation of shared decision making: An international perspective. Z. Evidenz Fortbild. Qual. Gesundh..

[7] National Institute for Health and Care Excellence (NICE) (2016). Shared Decision Making Collaborative. A Consensus Statement. https://www.nice.org.uk/Media/Default/About/what-we-do/SDM-consensus-statement.pdf.

[8] Mulley A.G., Trimble C., Elwyn G. (2012). Stop the silent misdiagnosis: Patients’ preferences matter. BMJ.

[9] Wennberg J.E. (2004). Practice variation: Implications for our health care system. Manag. Care.

[10] Stacey D., Légaré F., Lewis K., Barry M.J., Bennett C.L., Eden K.B., Holmes-Rovner M., Llewellyn-Thomas H., Lyddiatt A., Thomson R. (2017). Decision aids for people facing health treatment or screening decisions. Cochrane Database Syst. Rev..

[11] Brody D.S. (1980). The patient’s role in clinical decision-making. Ann. Intern. Med..

[12] Härter M., Moumjid N., Cornuz J., Elwyn G., van der Weijden T. (2017). Shared decision making in 2017: International accomplishments in policy, research and implementation. Z. Evidenz Fortbild. Qual. Gesundh..

[13] Josefsson K.A., Andersson A.C. (2017). The co-constructive processes in physiotherapy. Cog. Med..

[14] Dierckx K., Deveugele M., Roosen P., Devisch I. (2013). Implementation of shared decision making physical therapy: Observed level of involvement and patient preference. Phys. Ther..

[15] Sam S., Sharma R., Corp N., Igwesi-Chidobe C., Babatunde O.O. (2020). Shared decision making in musculoskeletal pain consultations in low-and middle-income countries: A systematic review. Int. Health.

[16] Joseph-Williams N., Lloyd A., Edwards A., Stobbart L., Tomson D., Macphail S., Dodd C., Brain K., Elwyn G., Thomson R. (2017). Implementing shared decision making in the NHS: Lessons from the MAGIC programme. BMJ.

[17] Kidd M.O., Bond C.H., Bell M.L. (2011). Patients’ perspectives of patient-centredness as important in musculoskeletal physiotherapy interactions: A qualitative study. Physiotherapy.

[18] Stenner R., Palmer S., Hammond R. (2018). What matters most to people in musculoskeletal physiotherapy consultations? A qualitative study. Musc. Sci. Pract..

[19] Stenner R., Swinkels A., Mitchell T., Palmer S. (2016). Exercise prescription for non-specific chronic low back pain (NSCLBP): A qualitative study of patients’ experiences of involvement in decision making. Physiotherapy.

[20] Ali N., May S. (2017). A qualitative study into Egyptian patients’ satisfaction with physiotherapy management of low back pain. Physiother. Res. Int..

[21] Hausheer A.C., Suter L.C., Kool J. (2020). Shared decision-making in physical therapy: A cross-sectional observational study. Eur. J. Physiother..

[22] Sutton A., Clowes M., Preston L., Booth A. (2019). Meeting the review family: Exploring review types and associated information retrieval requirements. Health Inf. Libr. J..

[23] Thomas J., Harden A. (2008). Methods for the thematic synthesis of qualitative research in systematic reviews. BMC Med. Res. Methodol..

[24] Maxwell J.A. (2012). A Realist Approach for Qualitative Research.

[25] Duncan E.A., Nicol M.M. (2004). Subtle realism and occupational therapy: An alternative approach to knowledge generation and evaluation. Br. J. Occup. Ther..

[26] Tong A., Flemming K., McInnes E., Oliver S., Craig J. (2012). Enhancing transparency in reporting the synthesis of qualitative research: ENTREQ. BMC Med. Res. Methodol..

[27] Pawson R., Greenhalgh T., Harvey G., Walshe K. (2005). Realist review-a new method of systematic review designed for complex policy interventions. J. Health Serv. Res. Policy.

[28] Salvador-Oliván J.A., Marco-Cuenca G., Arquero-Avilés R. (2019). Errors in search strategies used in systematic reviews and their effects on information retrieval. J. Med. Libr. Assoc..

[29] McGowan J., Sampson M., Salzwedel D.M., Cogo E., Foerster V., Lefebvre C. (2016). PRESS peer review of electronic search strategies: 2015 guideline statement. J. Clin. Epidemiol..

[30] Schoeb V., Bürge E. (2012). Perceptions of patients and physiotherapists on patient participation: A narrative synthesis of qualitative studies. Physiother. Res. Int..

[31] Joseph-Williams N., Elwyn G., Edwards A. (2014). Knowledge is not power for patients: A systematic review and thematic synthesis of patient-reported barriers and facilitators to shared decision making. Patient Educ. Couns..

[32] Cooke A., Smith D., Booth A. (2012). Beyond PICO: The SPIDER tool for qualitative evidence synthesis. Qual. Health Res..

[33] Eakin J.M., Mykhalovskiy E. (2003). Reframing the evaluation of qualitative health research: Reflections on a review of appraisal guidelines in the health sciences. J. Eval. Clin. Pract..

[34] Tong A., Sainsbury P., Craig J. (2007). Consolidated criteria for reporting qualitative research (COREQ): A 32-item checklist for interviews and focus groups. Int. J. Qual. Health Care.

[35] Thorne S., Jensen L., Kearney M.H., Noblit G., Sandelowski M. (2004). Qualitative metasynthesis: Reflections on methodological orientation and ideological agenda. Qual. Health Res..

[36] Moher D., Liberati A., Tetzlaff J., Altman D.G., Prisma Group (2009). Preferred reporting items for systematic reviews and meta-analyses: The PRISMA statement. PLoS Med..

[37] Payton O.D., Nelson C.E., Hobbs M.S. (1998). Physical therapy patients’ perceptions of their relationships with health care professionals. Physiother. Theory Pract..

[38] Cooper K., Smith B.H., Hancock E. (2008). Patient-centredness in physiotherapy from the perspective of the chronic low back pain patient. Physiotherapy.

[39] Potter M., Gordon S., Hamer P. (2003). The physiotherapy experience in private practice: The patients’ perspective. Aus. J. Physiother..

[40] Wikman A., Fältholm Y. (2006). Patient empowerment in rehabilitation: “Somebody told me to get rehabilitated”. Adv. Physiother..

[41] Lindahl M., Teljigović S., Heegaard Jensen L., Hvalsoe B., Juneja H. (2016). Importance of a patient-centred approach in ensuring quality of post-fracture rehabilitation for working aged people: A qualitative study of therapists’ and patients’ perspectives. Work.

[42] Bernhardsson S., Larsson M.E., Johansson K., Öberg B. (2017). “In the physio we trust”: A qualitative study on patients’ preferences for physiotherapy. Physiother. Theory Pract..

[43] Bernhardsson S., Samsson K.S., Johansson K., Öberg B., Larsson M.E. (2019). A preference for dialogue: Exploring the influence of patient preferences on clinical decision making and treatment in primary care physiotherapy. Eur. J. Physiother..

[44] Doyle L.H. (2003). Synthesis through meta-ethnography: Paradoxes, enhancements, and possibilities. Qual. Res..

[45] Caress A.L., Luker K., Woodcock A., Beaver K. (2002). A qualitative exploration of treatment decision-making role preference in adult asthma patients. Health Expect..

[46] Bastiaens H., Van Royen P., Pavlic D.R., Raposo V., Baker R. (2007). Older people’s preferences for involvement in their own care: A qualitative study in primary health care in 11 European countries. Patient Educ. Couns..

[47] Aasen E.M., Kvangarsnes M., Heggen K. (2012). Perceptions of patient participation amongst elderly patients with end-stage renal disease in a dialysis unit. Scand. J. Caring Sci..

[48] Frosch D.L., May S.G., Rendle K.A., Tietbohl C., Elwyn G. (2012). Authoritarian physicians and patients’ fear of being labeled ‘difficult’among key obstacles to shared decision making. Health Aff..

[49] Say R., Murtagh M., Thomson R. (2006). Patients’ preference for involvement in medical decision making: A narrative review. Patient Educ. Couns..

[50] Kelsey J., Abelson-Mitchell N., Skirton H. (2007). Perceptions of young people about decision making in the acute healthcare environment. Nurse Child. Young People.

[51] Patel S., Ngunjiri A., Sandhu H., Griffiths F., Thistlewaite J., Brown S., Friede T., Lord J., Tysall C., Woolvine M. (2014). Design and development of a decision support package for low back pain. Arthritis Care Res..

[52] Makoul G., Arntson P., Schofield T. (1995). Health promotion in primary care: Physician-patient communication and decision making about prescription medications. Soc. Sci. Med..

[53] Joseph-Williams N., Williams D., Wood F., Lloyd A., Brain K., Thomas N., Prichard A., Goodland A., McGarrigle H., Sweetland H. (2019). A descriptive model of shared decision making derived from routine implementation in clinical practice (‘Implement-SDM’): Qualitative study. Patient Educ. Couns..

[54] Pinto R.Z., Ferreira M.L., Oliveira V.C., Franco M.R., Adams R., Maher C.G., Ferreira P.H. (2012). Patient-centred communication is associated with positive therapeutic alliance: A systematic review. J. Physiother..

[55] Flynn K.E., Smith M.A., Vanness D. (2006). A typology of preferences for participation in healthcare decision making. Soc. Sci. Med..

[56] Chewning B., Bylund C.L., Shah B., Arora N.K., Gueguen J.A., Makoul G. (2012). Patient preferences for shared decisions: A systematic review. Patient Educ. Couns..

[57] Doherty C., Doherty W. (2005). Patients’ preferences for involvement in clinical decision-making within secondary care and the factors that influence their preferences. J. Nurs. Manag..

[58] Thompson A.G. (2007). The meaning of patient involvement and participation in health care consultations: A taxonomy. Soc. Sci. Med..

[59] Hargraves I., Montori V.M. (2014). Decision aids, empowerment, and shared decision making. BMJ.

[60] Ajzen I. (1991). The theory of planned behavior. Organ. Behav. Hum. Decis. Process..

[61] Pound P., Campbell R. (2015). Exploring the feasibility of theory synthesis: A worked example in the field of health related risk-taking. Soc. Sci. Med..

[62] Turner J.H. (1991). Developing cumulative and practical knowledge through metatheorizing. Sociol. Perspect..

[63] Hellmann G. (2003). In conclusion: Dialogue and synthesis in individual scholarship and collective inquiry. Int. Stud. Rev..

[64] Mays N., Pope C. (2000). Assessing quality in qualitative research. BMJ.

[65] Berger R. (2015). Now I see it, now I don’t: Researcher’s position and reflexivity in qualitative research. Qual. Res..

[66] Elwyn G., Frosch D., Thomson R., Joseph-Williams N., Lloyd A., Kinnersley P., Cording E., Tomson D., Dodd C., Rollnick S. (2012). Shared decision making: A model for clinical practice. J. Gen. Int. Med..

