# Supplementary material for: People’s Experience of Shared Decision Making in Musculoskeletal Physiotherapy: A Systematic Review and Thematic Synthesis
Source: Behav Sci (Basel). 2022 Jan 12;12(1):12. doi: 10.3390/bs12010012 (PMC8773142; doi:10.3390/bs12010012)
Supplement: Supplementary file 1 [file behavsci-12-00012-s001.zip › File S1 Search strategy audit trail.pdf]

Limits:

- Date not limited as no known systematic reviews have been done to date on this subject.
- Search terms not limited to musculoskeletal (MSK) physiotherapy even though this was part of the inclusion criteria, as a scoping literature search showed that some titles and abstracts that were relevant did not stipulate the speciality of MSK in title or key words, and were being missed in the search.
- Search terms not limited to patient/client/service user/person as a scoping literature search showed this was not always represented in the title or keywords, and relevant articles were being missed.
- Search was limited to English language only.

Inclusion criteria were manually searched for in title/abstract/full text screening stages:

- S: individuals who had experience of undertaking MSK physiotherapy. Studies relating to other areas of healthcare and physiotherapy specialities were excluded.
- PI: studies must include at least 1 paragraph of explicit reference to SDM within the aim, objectives or results. Articles were accepted if this was contained within broader phenomena such as person centred care or satisfaction with care.
- D: a broad range of design types were included; all except conference proceedings and pilot studies were included.
- E: exploration of people's experiences of SDM in MSK physiotherapy. Studies must report the first hand experiences or perceptions of people received physiotherapy treatment, as opposed to perceptions of healthcare professionals.
- R: Qualitative, quantitative and mixed methods research is all included, whilst pilot studies and conference proceedings are excluded.
- Other: Date of publication was not limited, as a scoping literature search demonstrated no known Systematic Reviews published previously to update. Language was limited to English as the primary language understood by the review team.

(Cook et al., 2012)

Record of searches performed:

- Ebsco host: AMED, CINAHL, MEDLINE/OVID: 1028 results

Shared N2 decision N2 making **OR** Person N2 centred N2 care **OR** Patient N2 centred N2 care

AND physio*

- EMBASE: 440 results

Shared adj decision adj making **OR** person adj centred adj care **OR** patient adj centred adj care **AND** physio*

Duplicates: 10

- Cochrane: 22 results

The original search terms Shared decision making **OR** Person centred care **OR** Patient centred care **AND** physio* yielded no results. Using a broader 'Shared Decision Making' only yielded 22 results.

- Find it bham: 12 relevant after title screening

As with the Cochrane database, search terms were kept broad; Shared decision making, physiotherapy. This yielded many results, but the authors stopped reviewing titles after 110 results as the articles were becoming increasingly irrelevant.

- Google scholar: 0

As with the Cochrane database, search terms were kept broad; Shared decision making, physiotherapy. This yielded many results, but the authors stopped reviewing titles after 240 results as the articles were becoming increasingly irrelevant. No new relevant articles were found

- Reference list checking: 5

Targeted checking of the included articles' reference lists. This resulted in 5 new articles eligible for screening.

- Grey literature

CSP website, used search box with 'shared decision making', which yielded 7128 results. No extra articles were included in line with the eligibility criteria, and the authors stopped reviewing titles after the first 60 as they were becoming increasingly irrelevant.

Choosing wisely UK https://www.choosingwisely.co.uk/shared-decision-making/ no extra articles included in line with the eligibility criteria.

Key authors

Glyn Elwyn: http://www.glynelwyn.com/ no extra articles included in line with the eligibility criteria.

Searched Natalie Joseph-Williams and France Legare on google scholar, no extra articles included in line with the eligibility criteria.

NICE: used search box with ‘shared decision making’ which yielded 421 results. Except from a Shared Decision Making guideline in process which is reportedly due in April 2021, there were no relevant studies, and therefore no extra articles were included in line with the eligibility criteria.
