# Supplementary material for: People’s Experience of Shared Decision Making in Musculoskeletal Physiotherapy: A Systematic Review and Thematic Synthesis
Source: Behav Sci (Basel). 2022 Jan 12;12(1):12. doi: 10.3390/bs12010012 (PMC8773142; doi:10.3390/bs12010012)
Supplement: Supplementary file 1 [file behavsci-12-00012-s001.zip › File S2 Critical appraisal discussions.pdf]

Critical appraisal discussions

Process: all of the included articles were discussed in light of their COREQ scores, limitations and perspectives that they brought to the review. Particular criticality was applied to articles that scored less than 20.

The authors discussed what they felt was pertinent to quality outside of the COREQ, and came up with the following:

- **Considerations to reflexivity**

Consistent across studies was a lack of documented reflexivity. However, because the interest in development of data, themes and saturation was the main focus of studies contributing to this review, the importance of this section was reduced for the purpose of this synthesis.

- **Consideration of methods, data analysis and synthesis.**

Instead, more weighting was put on considerations for studies transparency in reporting their methods, data analysis and synthesis, as well as consideration of plausibility of their findings. Eg. Are the results believable? Are they anomalous to the other studies as a result of methods or analysis/synthesis flaws?

- **Important points around data and what it achieves as a review criteria.**

Consideration was also made for how integral the studies' results were in relation to creating new perspectives within this review. Eg. Payton et al., (1998) scored lowest on COREQ but the findings gave the greatest challenge to results.

Potter et al., (2003)

19/32 on COREQ

- Nominal group technique – variant
- Sample size was good, confidence in common themes
- Piloted
- Data saturation considered
- Multiple coders so agreement on themes would have been a consideration

Given the above points of strength Potter was kept in.

Payton et al., (1998)

<16 COREQ – lowest scoring study

- Part of methods reporting and selection of participants wasn't clear -
- Participant drop out rate and reasons not given -
- They don't discuss data saturation or member checking -

Audio recording +

- Piloted data +
- Consistent application of interviews +

Results that came out provided a different perspective to the other included studies because it considered participant demographics and characteristics as directly impacting

people's preferences. It provides a new perspective, but the level of rigor within the study is questionable. Overall, the results are unique, and therefore give a positive addition to the review. It was therefore agreed to keep Payton et al., (1998) in.

Lindahl et al., (2016)
25/32 COREQ

- Sampling unclear
- Developed a guide which was piloted first and audio recorded
- Discussed data saturation
- Description of the coding tree

Deemed a good study both qualitatively and by the COREQ.

Cooper et al., (2008)
22/32 COREQ

- Interview guide not pilot tested
- Field notes not documented
- Multiple coders
- Explicit about data analysis process (used NVIVO)
- Coding tree identified
- Good reporting of study

Happy that the negative aspects of this study would not cause concern for the inclusion.

Wilkman and Faltholm, (2006)
17/32 COREQ

- Pilot testing unclear
- Data saturation discussed
- Two coders which infers good discussions around at analysis
- Provided a description of the coding tree
- Data was a different perspective – mixed inpatients and outpatients, in inpatients there was didactic approach – so valuable insight given compare to other studies.

Minor points are interview schedule development. It provided support for the synthesis of this review and novel insight, so it was agreed that Wilkman and Faltholm, (2006) should be included.

Ali and May, 2017
26/32 COREQ

- Egyptian study
- Cultural perspective given
- Participant dropout wasn't clear
- They developed interview schedule

- Two coders

No problems with the study.

Stenner et al., (2016)
16/32 COREQ

- Scored low on reflexivity
- Sampling documented
- Unclear about field notes
- Did document duration of interview
- No discussion of data saturation

British study looking at lower back pain, results and conclusions challenged the roles well and was critical of important aspects relating to the synthesis. Results highly valued therefore study included.

Bernhardsson et al., (2017)
28/32
Unclear if member checking
No description of coding tree

Strong study and therefore included.

Bernhardsson et al., (2019)
27/32
Didn't say where interviews carried out
unclear transcripts returned to participants

Strong study and therefore included.
