# Supplementary material for: People’s Experience of Shared Decision Making in Musculoskeletal Physiotherapy: A Systematic Review and Thematic Synthesis
Source: Behav Sci (Basel). 2022 Jan 12;12(1):12. doi: 10.3390/bs12010012 (PMC8773142; doi:10.3390/bs12010012)
Supplement: Supplementary file 1 [file behavsci-12-00012-s001.zip › File S3 Analysis, synthesis and refinement.pdf]

- Stage 1: identification of study characteristics and participant demographics (shown in Table 1)
- Stage 2: Line by line coding
- Stage 3: development of descriptive themes (see Appendix 2)
- Stage 4: Development of analytical themes (see Appendix 3)
- Stage 5: integration and refinement

The following is an audit trail of the cognitive processes between Stage 4 and stage 5

Key: Black text is original analytical themes
Blue text shows cognitive processes during review of analytical themes
Green text shows the finalised themes following integration and refinement

Stage 4: Development of analytical themes

Theme 1: Trust

Sub theme A; People trust physiotherapists, and this has implications on decision making

- The 'therapist knows best' (8). People were happy to defer decisions to the 'expert' clinician (1, 5), and were happy they would make the 'right' decision (1, 6). This may be influenced by the fear of making the 'wrong' decision if left to be completely autonomous.
- Trust was fostered by perceived passion, personal competence, communication skills, taking the person seriously and empathic personality traits (1, 2, 3, 5, 9).
- Regardless of the preference for DM, trust resulted in a positive experience, and could be mutual (8). It improved engagement (1) and reduced fear (8, 9).

Sub theme B; Trust can hamper people's involvement in decision making

- Trust in the expert was cited as a reason for someone to defer a decision, and therefore defer their own involvement (1, 2, 3, 4, 5, 8)
- People felt the physio would choose what was best (2, 3, 8), especially if not sure on their own preferences (2).
- Trust could be fostered solely because the physio was deemed an 'expert' (2, 3, 6).
- Perceived 'expertise' was not always welcomed and 'know it all physios (8), misunderstanding people's preferences led to the physio blocking the person's ability to participate (8).

Stage 5: Integration and refinement

Re-reading the themes, I think the subthemes could be split more appropriately into A how trust is developed, B The positive impact of trust and C The negative impact of trust. This was influenced by a discussion on refinement with the second author AS.

Theme 1: Trust

Sub theme A: how is trust developed?

- Trust was fostered by perceived passion, personal competence, communication skills, taking the person seriously and empathic personality traits (1, 2, 3, 5, 9).
- Trust could be fostered solely because the physio was deemed an ‘expert’ (2, 3, 6).

Sub theme B: The positive impact of trust

- Regardless of the preference for DM, trust resulted in a positive experience, and could be mutual (8). It improved engagement (1) and reduced fear (8, 9).
- People felt the physio would choose what was best (2, 3, 8), especially if not sure on their own preferences (2).

Sub theme C: The negative impact of trust

- The ‘therapist knows best’ (8). People were happy to defer decisions to the ‘expert’ clinician (1, 5), and were happy they would make the ‘right’ decision (1, 6). This may be influenced by the fear of making the ‘wrong’ decision if left to be completely autonomous.
- Trust in the expert was cited as a reason for someone to defer a decision, and therefore defer their own involvement (1, 2, 3, 4, 5, 8).
- Perceived ‘expertise’ was not always welcomed and ‘know it all physios (8), misunderstanding people’s preferences led to the physio blocking the person’s ability to participate (8).

Stage 4: Development of analytical themes

Theme 2: treatment preferences

Sub theme A; People's preferences for treatments vary, but preferences should influence treatment decisions

- Peoples' preferences for treatments vary (1, 5, 7); some want active strategies, some want passive treatment (1). This is based on their own experience (social learning theories).
- People felt their preferences for treatment should be taken into account (1); when they weren't this was negative and even resulted in reduced 'compliance' with a programme. Not understanding peoples' preferences led to perception of generic treatments, therefore treatment should be tailored to improve satisfaction (2, 4, 5).
- There can be mismatch in people's expectations for treatment and what they are given (4).

Sub theme B; People want treatments to be based on evidence

- Some people felt treatment should be evidence based, and that if the physio kept up to date with their own knowledge, this could translate into good decision making for treatment choices (1,7).

Sub theme C; People want treatments to be tailored and make sense

- People want treatments to be individualised and tailored (5)
- Tailored treatment was perceived to happen when good communication around the explanation occurred (5) The inference here then is if clinicians do this, people will feel their Rx is individualised, makes more sense and more likely to have +ve experience.
- If exercises were prescribed which didn't take into account someone's' preferences, they were less likely to be done (5). Could this fit with the -ve impact of when preferences aren't taken into account? (one half of column C).

Stage 5: Integration and refinement

Theme 2: treatment preferences

After reading through the original sub themes, it appeared that there was clearer sub themes of the positive impact of when treatment preferences inform treatment, and the negative impact when the opposite happened, spread throughout the original 3 sub themes. I think it makes more sense to relabel and reorganise the content here. The original 1st sub theme is preserved with the addition of 'individual factors' to encompass the individual nature of preferences. The second half of sub theme A is split out into it's own new sub theme. The refined sub themes are as follows;

Sub theme A; People's treatment preferences vary due to individual factors

- Peoples' preferences for treatments vary (1, 5, 7); some want active strategies, some want passive treatment (1). This is based on their own experience (social learning theories).
- Some people felt treatment should be evidence based, and that if the physio kept up to date with their own knowledge, this could translate into good decision making for treatment choices (1,7).
- People want treatments to be individualised and tailored (5).
- Tailored treatment was perceived to happen when good communication around the explanation occurred (5) The inference here then is if clinicians do this, people will feel their Rx is individualised, makes more sense and more likely to have +ve experience.

Sub theme B; The impact of when preferences influence treatment

- People felt their preferences for treatment should be taken into account (1).
- Treatment should be tailored to improve satisfaction (2, 4, 5).
- People want treatments to be individualised and tailored (5).
- Influencing the decision made people feel treatment was tailored to their needs (1).

Sub theme C; The impact of when preferences do not influence treatment

- When treatments weren't based on people's preferences this was negative and even resulted in reduced 'compliance' with a programme. Not understanding peoples' preferences led to perception of generic treatments (2, 4, 5).
- If exercises were prescribed which didn't take into account someone's' preferences, they were less likely to be done (5).

After consideration, and due to the word count restrictions which govern systematic reviews, it was decided to take out 'Decision preferences' from the results and discussion section of the review. Whilst it has shown valuable insight into ideas such as concordance to rehabilitation programme sin MSK physiotherapy, it was felt that these findings were the least impactful when compared with the other data. It was also felt that removal of these findings would have the least impact on overall findings and discussion of the wider review.

Stage 4: Development of analytical themes

Theme 3: Decision preferences

Sub theme A; People's preferences for decision making varies

- Preference for how much the person was involved in a decision varied (1, 2, 4, 5, 8), some people were passive, some were more active, and lots were in the middle (1, 8).
- Preference for how much involvement they had could vary based on each decision, and this could be due to the perceived level of risk the decision could bring (4,8).
- People wanted to be involved, even if they didn't make the actual decision (1, 2) (I think this data fits better with the sub theme, people want to be involved).
- In terms of satisfaction with involvement, whether they were involved or not, some people felt they wouldn't want DM to change, whilst others showed ambiguity with their involvement. Shows collaboration with DM is complex (8).
- Statistical analysis showed factors which might influence DM preferences were cultural, social and economic ones (8); only limited understanding of this due to stats, but could be a starting point for future research. I think this point fits better with the above point of factors which could influence DM preference.

Sub theme B; People want to be involved in decision making

- People want to be involved in decision making throughout the process (1,2, 3, 8), including goal setting and treatment choice (8).
- People wanted their preferences to be taken into account (6,8) and they want choices (5). When this happens, they feel they have gained control (9).
- Some people are very empowered to collaborate and make decisions (1). Someone said if they could have made all the decisions they would have (8), which infers some outside entity blocking their ability to make decisions. This also fits with Theme 4, sub theme A
- Influencing the decision made people feel treatment was tailored to their needs (1) I think this fits better with Theme 2, sub theme B.

Sub theme C; People don't want to be involved in decision making

- People were happy to defer decisions to the physio (1, 2, 3, 4, 5).
- People felt it was the physios' role to make the decision in the best interest of the person, and the person's role to listen to them (1, 3, 5, 8).
- People were happy to defer because the physio was the expert who knows what is best, and knows the persons' preferences (1, 3, 4, 5) because of fear of making the wrong decision (3,4), if the explanation was good (5), if the decision was 'minor' (4) and if they did not think it was their role to make decisions (4, 5, 8).
- Less educated group less likely to be involved (8). This isn't clear if it is a preference or not so could be moved over to next column as the inference here is that the less educated may be affected by the capacity to participate in DM.

Stage 5: integration and refinement

After reviewing Theme 3, I feel that the sub themes are appropriately named. However some of the data required shifting to other sub themes to result in a better fit and understanding (see text in light blue for cognitive process).

Theme 3: Decision preferences

Sub theme A; People's preferences for decision making varies

- Preference for how much the person was involved in a decision varied (1, 2, 4, 5, 8), some people were passive, some were more active, and lots were in the middle (1, 8).
- Preference for how much involvement they had could vary based on each decision, and this could be due to the perceived level of risk the decision could bring (4,8), but one study showed it could be due to cultural, social and economic factors (8); only limited understanding of this due to method of data collection (statistical analysis).
- In terms of satisfaction with involvement, whether they were involved or not, some people felt they wouldn't want DM to change, whilst others showed ambiguity with their involvement. Shows collaboration with DM is complex (8).

Sub theme B; People want to be involved in decision making

- People want to be involved in decision making throughout the process (1, 2, 3, 8), including goal setting and treatment choice (8), and even if they don't make the actual decision (1).
- People wanted their preferences to be taken into account (6, 8) and they want choices (5). When this happens, they feel they have gained control (9).
- Some people are very empowered to collaborate and make decisions (1). Someone said if they could have made all the decisions they would have (8), which infers some outside entity blocking their ability to make decisions.

Sub theme C; People don't want to be involved in decision making

- People were happy to defer decisions to the physio (1, 2, 3, 4, 5).

- People felt it was the physios' role to make the decision in the best interest of the person, and the person's role to listen to them (1, 3, 5, 8).
- People were happy to defer because the physio was the expert who knows what is best, and knows the persons' preferences (1, 3, 4, 5) because of fear of making the wrong decision (3, 4), if the explanation was good (5), if the decision was 'minor' (4) and if they did not think it was their role to make decisions (4, 5, 8).

Stage 4: Development of analytical themes

Theme 4: Decision ability

Sub theme A; People aren't involved in decision making

- Physio laid out options, and then proffered the one they thought best. This means the person isn't involved equally in the DM (1).
- Physio chose the treatment independently of the person (2, 4, 6, 8).
- There were no treatment options laid out (2, 5), or not all of the options were laid out which could lead to perceived inequity (5).
- The physio did not take into account the persons' preferences for treatment decision, and this led to a negative experience (in some cases an emotionally) and could affect compliance (5, 7, 8).
- Physios exerted 'power' over the relationship (7), people were told what to do (4), and if this opposed the person's beliefs, could impact the physio experience negatively (7, 5, 4). This could lead to disempowerment. I think this fits better with sub theme B, knowledge and experience is power.

Sub theme B; The power struggle

- Previous experience of physio helped people to understand what worked for them and left them empowered (1).
- More knowledge resulted in a greater ability to participate in DM or even to self-manage (2, 3, 4, 5), and knowledge could be gained from information provision from the therapist (2), but only if it was presented in non-medical terms (5). This is close to the 'people want information' but has bigger implications as to why people want info, to enable them. Lack of knowledge left people unable to challenge the physio (4), and a lack of knowledge meant they didn't know how to help themselves (4).
- Positive previous experience led to increased self-confidence (2), whereas lack of confidence led people to feel they couldn't challenge the physio when they wanted more involvement (4).
- The ability to ask questions and challenge could come from previous experience (2, 7) knowledge (2, 3) and confidence (4). People felt they would like to have been involved in decisions within their abilities (8).

Stage 5: Integration and refinement

Theme 4: Decision ability

After reviewing Theme 4 including the sub themes, I think the labelling and content is sound. There was one change within the content which was movement of one point around physiotherapist power from sub theme A to sub theme B. I have also added a point from Theme 6 sub theme A, to Theme 4 Sub theme A, as it fits well with this category.

Sub Theme A; People aren't involved in decision making

- Physio laid out options, and then proffered the one they thought best (1), or the physio chose the treatment completely independently of the person (2, 4, 6, 8). This means the person isn't involved equally in the DM.
- Other times, there were no or only some treatment options laid out (2, 5), which could lead to perceived inequity (5).
- Someone said if they could have made all the decisions they would have (8), and id they had more input, they would have worked a lot harder (8), which infers some outside entity blocking their ability to make decisions.
- The physio did not take into account the persons' preferences for treatment decision, and this led to a negative experience (in some cases an emotionally) and could affect compliance (5, 7, 8).

Sub theme B; The power struggle

- Previous experience of physio helped people to understand what worked for them and left them empowered (1).
- More knowledge resulted in a greater ability to participate in DM or even to self-manage (2, 3, 4, 5), and knowledge could be gained from information provision from the therapist (2), but only if it was presented in non-medical terms (5). This is close to the 'people want information' but has bigger implications as to why people want info, to enable them. Lack of knowledge left people unable to challenge the physio (4), and a lack of knowledge meant they didn't know how to help themselves (4).
- Positive previous experience led to increased self-confidence (2), whereas lack of confidence led people to feel they couldn't challenge the physio when they wanted more involvement (4).
- The ability to ask questions and challenge could come from previous experience (2, 7) knowledge (2, 3) and confidence (4). People felt they would like to have been involved in decisions within their abilities (8).
- Physios exerted 'power' over the relationship (7), people were told what to do (4), and if this opposed the person's beliefs, could impact the physio experience negatively (7, 5, 4). This could lead to disempowerment.

Stage 4: Development of analytical themes

Theme 5: Communication

Sub theme A; People want information

- People want information on diagnosis, prognosis, treatment options and self-management strategies (1,2, 3, 4, 5, 7, 9).
- Being offered information can lead to a more positive experience (2, 5, 7), the ability to have fears allayed (4) and empowerment to make informed decisions (2, 3).
- Information should be presented in a way people can understand (9), if it isn't this can lead to a negative experience (3, 5, 6, 7). Information available isn't always sufficient (4).

Sub theme B; People want to be listened to

- People felt it was integral to be listened to and have their preferences heard (1, 2, 8, 9) even if their preference was divergent to the physio (1), and decisions shouldn't be made without being listened to (1).
- This not only led to a more positive experience (2, 5, 7, 9) and the ability to develop trust (2), but people felt that part of their role as the patient was to help the physio create goals and treatment based on the feedback and information they provided (1, 2, 8).
- Feeling listened to, or a physio demonstrating empathy, resulted in greater satisfaction and trust (2, 5,7) and not being listened to resulted in frustration (5, 8).
- 1:1 physio resulted in people feeling that their preferences were heard more than group based intervention.

Sub theme C; Two way communication is essential for collaboration and for SDM

- Being listened to enabled people to participate in DM (2) and scenarios that actively encouraged questions and feedback further facilitated this (2, 5).
- 2 way communication between people/patients is helpful in group based intervention (5).
- Collaborative communication is integral for a good experience and a person's satisfaction (7), poor, didactic communication leads to a negative experience (7, 8).

Stage 5; Integration and refinement

Theme 5: Communication

- Change the sub theme A to 'People want *appropriate* information'.
- Rename sub theme C to 'Two way communication is essential for collaboration'.

There are positive and negative categories that come out of sub theme A, B and C, so I have considered whether the sub themes need to be relabelled as A; the positive impact of good communication, B; the negative impact of poor communication, and C; two way communication is needed for collaboration. However, the integral information that came out of the studies was the value placed on two way communication. I think the original sub themes encompass wanting information provision from physio to person, and wanting to be listened to, from person back to physio fits with this. I have therefore chosen to keep the original sub themes intact, with a slight change to some of the wording.

Sub theme A; People want appropriate information

- People want information on diagnosis, prognosis, treatment options and self-management strategies (1,2, 3, 4, 5, 7, 9).
- Being offered information can lead to a more positive experience (2, 5, 7) , the ability to have fears allayed (4) and empowerment to make informed decisions (2, 3).
- Information should be presented in a way people can understand (9), if it isn't this can lead to a negative experience (3, 5, 6, 7). Information available isn't always sufficient (4).

Sub theme B; People want to be listened to

- People felt it was integral to be listened to and have their preferences heard (1, 2, 8, 9) even if their preference was divergent to the physio (1), and decisions shouldn't be made without being listened to (1)
- This not only led to a more positive experience (2, 5, 7, 9) and the ability to develop trust (2), but people felt that part of their role as the patient was to help the physio create goals and treatment based on the feedback and information they provided (1, 2, 8)
- Feeling listened to, or a physio demonstrating empathy, resulted in greater satisfaction and trust (2, 5, 7) and not being listened to resulted in frustration (5, 8).
- 1:1 physio resulted in people feeling that their preferences were heard more than group based intervention

Sub theme C; Two way communication is essential for collaboration

- Being listened to enabled people to participate in DM (2) and scenarios that actively encouraged questions and feedback further facilitated this (2, 5).
- 2 way communication between people/patients is helpful in group based intervention (5).
- Collaborative communication is integral for a good experience and a person's satisfaction (7), poor, didactic communication leads to a negative experience (7, 8).

Stage 4: Development of analytical themes

<u>Theme 6: Benefits of SDM</u>

Sub theme A; More involvement in decision making can have an impact on outcome

- Someone felt if they had more input, they would have worked a lot harder (8).

Stage 5: Integration and refinement

Upon review, I feel that this doesn't fit in a separate heading, as there are lots of other wide ranging benefits of SDM cited throughout the data which have been included in other themes.

Instead, I think it should be moved to 'people aren’t involved in decision making’. This means that Theme 6 can be erased as a standalone category.
