# Supplementary material for: People’s Experience of Shared Decision Making in Musculoskeletal Physiotherapy: A Systematic Review and Thematic Synthesis
Source: Behav Sci (Basel). 2022 Jan 12;12(1):12. doi: 10.3390/bs12010012 (PMC8773142; doi:10.3390/bs12010012)
Supplement: Supplementary file 1 [file behavsci-12-00012-s001.zip › File S4 Reflexivity bio.pdf]

Researcher’s background

Jessica Grenfell was born and grew up in England, UK. After completing her undergraduate Physiotherapy degree, followed by Junior Physiotherapy rotations in various NHS settings in Devon and Worcestershire, she chose to specialise in Musculoskeletal (MSK) Physiotherapy in the Aneurin Bevan University Health Board, South Wales. Her interests led her to undertake an MSc at the University of Birmingham in Advanced Manipulative Physiotherapy, and exposure to critical learning styles here sparked an interest in contributing to the development of knowledge and understanding through research. Following graduation she relocated to Cornwall.

Andrew Soundy completed his undergraduate degree and PhD at the University of Exeter. He moved to Birmingham in 2005 to work for the department of physiotherapy at the University. Since then his post-doctoral research has focused on psychosocial factors that influence individuals within health care settings. He has published several articles on shared decision making and values the importance of non-judgemental, non-directive and empowering therapeutic encounters.

Researcher’s viewpoint

Jessica Grenfell

Clinically, her interests lie in exploring how healthcare professionals can assist people to be more engaged in their healthcare journey, becoming more empowered and responsible users of healthcare. Part of this journey has resulted in experience with Shared Decision Making as an approach to person centred care. Since that time, Jessica has witnessed the benefits that collaboration can have for both clinician and service user, and is interested in exploring ways in which both parties can become more confident with such approaches. This interest led Jessica to embark upon a systematic review exploring peoples’ experiences of shared decision making in MSK physiotherapy.

Andrew Soundy

Most of Andrews early post-doctoral review-based studies have assumed a philosophical position of subtle-realism. As a result of this position many past qualitative reviews have developed models and theories. The aim of developing such theories is to achieve analytical generalisability. However, his most recent research has been associated with social constructivism and he has published guidance for how meta-ethnography can assume such a position.
