# Supplementary material for: People’s Experience of Shared Decision Making in Musculoskeletal Physiotherapy: A Systematic Review and Thematic Synthesis
Source: Behav Sci (Basel). 2022 Jan 12;12(1):12. doi: 10.3390/bs12010012 (PMC8773142; doi:10.3390/bs12010012)
Supplement: Supplementary file 1 [file behavsci-12-00012-s001.zip › Table S2 Study characteristics.pdf]

Bernhardsson S, Samsson KS, Johansson K, Öberg B, Larsson ME. A preference for dialogue: exploring the influence of patient preferences on clinical decision making and treatment in primary care physiotherapy. European Journal of Physiotherapy. 2019; 21(2):107-14.	Bernhardsson S, Larsson ME, Johansson K, Öberg B. " In the physio we trust" : A qualitative study on patients' preferences for physiotherapy. Physiotherapy theory and practice. 2017; 33(7):535-49.
Sweden	Sweden
To explore how people with MSK pains' preferences were accommodated in clinical decision making, and how these were influenced by their rehabilitation	To explore peoples' preferences for treatment and involvement in clinical decision making?
18 people attending physio in Sweden for back, neck or shoulder pain, with symptoms from 1 month up to 15+ years. Aged between 22-77 years old. 10 male, 10 female. Various educational backgrounds and working status.	20 people attending physio in Sweden for back, neck or shoulder pain, with symptoms from 1 month up to 15+ years. Aged between 22-77 years old. 10 male, 10 female. Various educational backgrounds and working status.
Primary care setting	Primary care setting
Participants purposively sampled. Semi structured interviews, inductive exploratory approach. Interview guide developed, tested in pilot beforehand. Qualitative content analysis used.	Participants purposively sampled. Semi structured interviews, inductive exploratory approach. Interview guide developed, tested in pilot beforehand. Qualitative content analysis used.
Overarching theme identified, A preference for dialogue: communication essential for collaborative rehabilitation. This was split into 4 sub categories; My views matter, Two-way communication, leaving decisions to the physio, and physiotherapy influences preferences. Most peoples' preferences had influenced treatment decisions, and most decisions were made jointly.	Overarching theme identified "Trust in the physiotherapist fosters active engagement in therapy" page 537. This was split into 6 sub categories. People preferences for treatment and involvement in decision making varied. People wanted information on risks/benefits and wanted their voices heard, regardless of how much they wanted to be involved in the treatment decision. Those who want to have less influence over treatment decisions were influenced by 'trust' in the physiotherapist. This trust was often fostered through previous or external influences, not necessarily with the individual physiotherapist.
27/32	28/32

Cooper K, Smith BH, Hancock E. Patient-centredness in physiotherapy from the perspective of the chronic low back pain patient. Physiotherapy. 2008; 94(3):244-52.	Stenner R, Swinkels A, Mitchell T, Palmer S. Exercise prescription for non-specific chronic low back pain (NSCLBP): a qualitative study of patients' experiences of involvement in decision making. Physiotherapy. 2016; 102(4):339-44.	Ali N, May S. A qualitative study into Egyptian patients' satisfaction with physiotherapy management of low back pain. Physiotherapy research international. 2017; (2):e1647.
UK	UK	Egypt
To define patient-centeredness, in the context of physiotherapy for chronic low back pain, from the patient's perspective	To explore the experiences of involvement in treatment decision making, and the information and decision support needs of people with low back pain	To explore patients' expectations and satisfaction with physiotherapy for low back pain
20 people attending physio for low back pain, 5 male, 20 female, symptoms duration unclear. Aged 18-65. Mixed employment status	8 people attending physio for low back pain, 4 female, 4 male, with symptoms from 1-40 years. Aged 18-75+ Educational background unclear, mixed employment status.	18 people attending physio for low back pain, 9 male, 9 female, with symptoms from <3 weeks to >20 years. Aged 19-81. Mostly higher education background, mixed working status.
NHS	NHS	University and private healthcare
Framework analysis approach. Patients purposively sampled, semi structured interviews used. Interview guide developed based on literature review.	Philosophical hermeneutic approach. Participants purposively sampled, semi structured interviews used.	Framework analysis approach. Patients purposively sampled, semi structured interviews used. Interview guide developed and pilot tested.
6 themes identified; communication, decision making, individual care, information sharing and the Physiotherapist. Communication was the most important dimension which underpinned all others, and was integral for patient centeredness.	4 themes identified; patients' expectations and patients' needs are not synonymous, informationis necessary but often not sufficient, not all decisions need to be shared, and wanting to be treated as an individual. Shared decision making did not appear to be happening, although this may not be what every patient wants.	5 key themes identified; Outcome, patient education, the therapist, service provision and decision making. The physiotherapeutic relationship between clinician and person is complex and satisfaction determinants are multifactorial. Preference for involvement in decision making varied.
22/32	16/32	26/32

Payton OD, Nelson CE, Hobbs MS. Physical therapy patients' perceptions of their relationships with health care professionals. Physiotherapy Theory and Practice. 1998; 14(4):211-21.	Potter M, Gordon S, Hamer P. The physiotherapy experience in private practice: the patients' perspective. Australian Journal of Physiotherapy. 2003; 49(3):195-202.	Melander Wikman A, Fältholm Y. Patient empowerment in rehabilitation:“ Somebody told me to get rehabilitated” . Advances in Physiotherapy. 2006; 8(1):23-32.
America	Australia	Sweden
To explore how people perceive their roles and relationships with healthcare professionals, with a focus on decision making.	To explore peoples' perspectives on 'good' physiotherapy, and their insight on characteristics of good and bad physiotherapy experiences.	To describe the patient’ s experiences and influence and participation in the rehabilitation process
109 people. Unclear age range, most were under 65, 59% male, 41% female, 84% Caucasian, 34% blue collar, 49% white collar, 87% earned over 12,000 PA, the US poverty line at the time. Unclear symptom duration.	26 participants; 39% were male, 62% were female. 20-79 years age range (mean of 48.8). 58% were married, 65% had post-secondary education with income up to 40000 Aus. dollars. 61% were private fee paying, 39% compensable. Unclear symptom duration.	6 people accessing healthcare for orthopaedic, neurological and/or orthopaedic conditions. 4 female, 2 male, aged between 35 and 58. Unclear education backgrounds and employment status. Unclear symptoms duration.
Unclear	Private	Primary care setting
Descriptive, comparative statistics used. Participants purposively sampled, interviews used.	Nominal group technique used and piloted previously. Participants purposively sampled.	Grounded Theory approach. Participants purposively sampled, semi structured interviews used.
On the whole, people need more information about what to expect going into physiotherapy. How people perceived their role, and how much they wanted to be involved in decision making varied greatly between groups, meaning physiotherapists need to tailor approaches to the individual.	Characteristics of a good physio experience were communication ability, professional behaviour and organisational ability, and characteristics of the service. Good communication was most important to a good experience, and bad experience was most often attributed to poor communication or poor service provisions. Communication was integral in order to involve people in their decisions.	Core category identified; the parallel process of rehabilitation. This was then split into 2 approaches; the traditional medical model which was based on peoples ‘ experiences of acute care and rehabilitation in hospital, and the individual model which was based on experiences of rehabilitation in primary care. The ability to participate in decision making varied dependent on which model was experienced.
12/32	19/32	17/32

Study	Lindahl M, Teljigović S, Heegaard Jensen L, Hvalsoe B, Juneja H. Importance of a patient-centred approach in ensuring quality of post-fracture rehabilitation for working aged people: a qualitative study of therapists' and patients' perspectives. Work. 2016; 55(4):831-9.
Country	Denmark
Research question	To identify factors that patients and therapists define as quality in rehabilitation for people post fractures
Population and characteristics	7 participants, 18-64 years olds undergoing physio following fractures. 5 male, 2 females, mixed employment status. 3 white collar, 3 blue collar and 1 business owner. Symptom duration 1-6 months
Healthcare setting	Mixed in patients and secondary care setting
Methods	Grounded Theory approach. Participants purposively sampled, semi structured interviews used.
Analysis	Core category identified as partnership, split into two categories of continuity of rehabilitation and patient centred approach. A patient centred approach was integral to developing partnership which was also informed by a biopsychosocial approach and professionalism. The person's perceived level of control was enhanced when treated holistically, when the approach was individualised and when they were involved in decision making.
COREQ score	25/32
